# A Fragile Balance: Managing Femoral Neck Fracture, Transient Osteoporosis, and COVID-19 in Late Pregnancy

**DOI:** 10.7759/cureus.70326

**Published:** 2024-09-27

**Authors:** Weng Liang Tan, Muhammad Fathi Hayyun, Juzaily F Leong, Rizal Abdul Rani, Nor Hamdan Mohamad Yahaya

**Affiliations:** 1 Orthopaedics and Traumatology, Universiti Kebangsaan Malaysia, Kuala Lumpur, MYS

**Keywords:** covid-19, covid-19 and pregnancy, fracture femoral neck, percutaneous screw fixation, transient osteoporosis of the hip (toh)

## Abstract

Transient osteoporosis of the hip (TOH) during pregnancy is a rare, self-limiting condition that frequently goes undiagnosed. However, if not managed properly, TOH can lead to significant complications, such as pathological fractures. We report a case of a 29-year-old primigravida at 33 weeks and four days of gestation who presented with a right femoral neck fracture following a fall. She had experienced prodromal hip pain for one month, initially misdiagnosed as pelvic girdle pain. Radiological evaluation revealed osteopenia and a sub-capital femoral neck fracture. Blood investigations were unremarkable. During hospitalisation, the patient was also diagnosed with asymptomatic COVID-19 infection, complicating the management approach. A multidisciplinary team decided on an elective caesarean section followed by closed manipulative reduction (CMR) and percutaneous screw fixation. Therefore, TOH in pregnancy requires timely diagnosis and intervention to prevent complications such as fractures. In cases complicated by concurrent conditions like COVID-19, multidisciplinary management is essential to ensure optimal outcomes for both mother and child.

## Introduction

Transient osteoporosis of the hip (TOH) had been described as early as 1959 by Curtiss and Kincaid as transitory demineralisation of the hip [1]. In 1968, Lequesne introduced the term “transient osteoporosis of the hip” in the literature [2]. TOH is a rare, self-limiting skeletal condition that commonly occurs in men in their fourth and fifth decades. Less frequently, it can also happen in young primigravidas who are in the third trimester of pregnancy or postpartum period. The incidence of TOH in pregnancy is approximately 0.4 per 100,000 pregnancies, although underreporting is likely [3].

The aetiology of TOH remains unclear, with the widely accepted hypothesis being femoral head vein dysfunction due to increased intraosseous pressure and venous hypertension [3]. Potential risk factors include hormonal changes, immobilisation, venous stasis, and mechanical pressure from the gravid uterus [4]. Clinically, TOH is a diagnosis of exclusion, ruling out the differential diagnoses, such as osteonecrosis of the femoral head (ONFH), fractures, infections, inflammatory arthritis, and malignancy.

Although rare, TOH in pregnancy can result in pathological fractures, with an estimated 12.1% of patients developing hip fractures [5]. To date, there have been many cases of TOH in pregnancy with femoral neck fractures reported. However, there are no established guidelines and consensus for the timing of delivery and orthopaedic surgery, options of implants, or delivery mode.

## Case presentation

**Figure 1:**
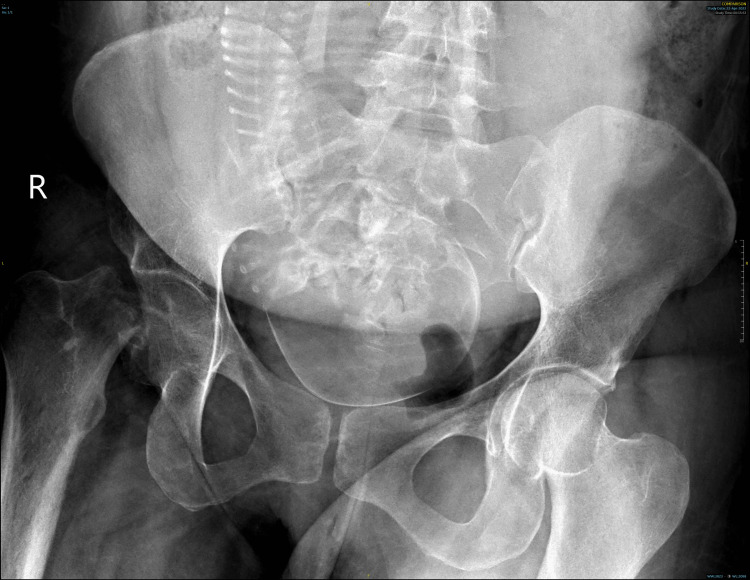
Pelvic X-ray post-trauma The X-ray shows a right femoral neck fracture with osteopenia of the right proximal femur and foetus in vertex presentation.

A 29-year-old primigravida, at 33 weeks and four days of gestation, presented with severe right hip pain and an inability to ambulate following a trivial fall on a slippery floor. On examination, the right lower limb was shortened, externally rotated, and flexed. X-rays showed a sub-capital fracture of the right femoral neck, with notable osteopenia in the proximal femur, including the head, neck, and trochanteric regions, compared to the contralateral side (Figure 1).

The patient denied a history of long-term steroid use, traditional medications, endocrine disorders, or prior infections or malignancy. She had not received any radiation to the pelvic or hip region. Her social history revealed that she was a non-smoker and did not consume alcohol. There was also no history of recreational drug use.

One month prior to this admission, the patient complained of spontaneous right hip pain without any trauma or injury. However, she was still able to ambulate. No blood and radiological investigations were done during the initial presentation. She was instead diagnosed with pregnancy-related pelvic girdle pain and was discharged with analgesics with no advice on weight-bearing restrictions.

On admission, her blood investigations were unremarkable, except for an elevated C-reactive protein (CRP). Further workup, including thyroid function tests, parathyroid hormone levels, serum calcium, vitamin D, serum and urine electrophoresis, and tumour markers, all returned within normal limits.

Unfortunately, the patient tested positive for asymptomatic (category 1) COVID-19 infection. A multidisciplinary team consisting of orthopaedic surgeons, obstetricians, gynaecologists, paediatricians, medical physicians, infectious disease physicians, and anaesthesiologists was convened to assess the risks and benefits of both orthopaedic and obstetric interventions. After a discussion with the patient and her husband, a decision was made to proceed with an elective lower segment caesarean section (LSCS) followed by closed manipulative reduction (CMR) and percutaneous screw fixation of the right femoral neck. Both the procedures were performed in the same setting on the seventh day of admission, following the completion of her COVID-19 quarantine according to the local protocol and the completion of corticosteroids with a gestational age of 34 weeks for foetal lung maturity.

**Figure 2:**
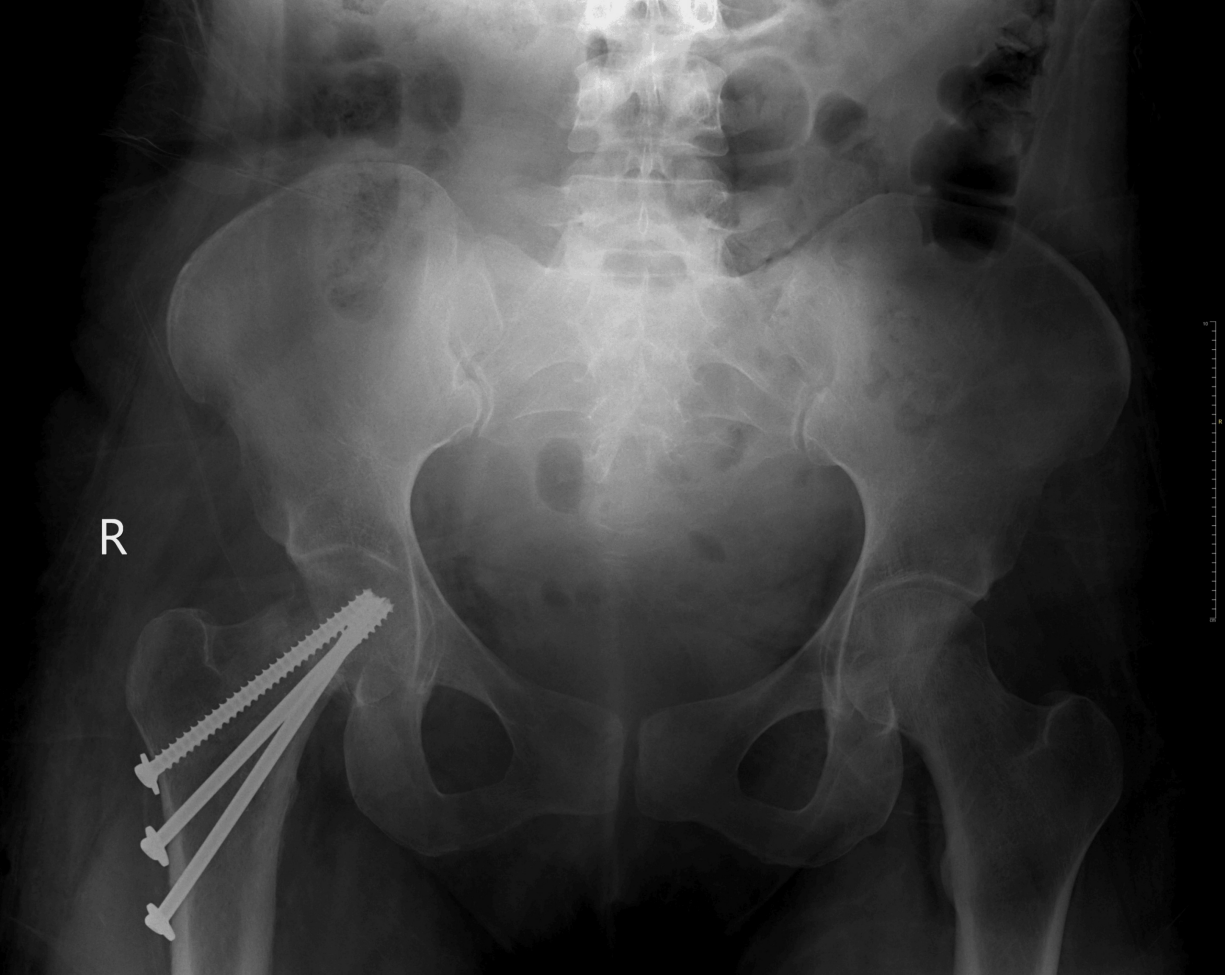
Post-screw fixation check X-ray The check X-ray shows acceptable fixation after percutaneous screw fixation of the right femoral neck.

The LSCS and the screw fixation were uneventful. Intra-operatively, a bone sample was sent for culture and sensitivity testing, which yielded no growth of organisms. Histopathological examination (HPE) of the bone was also normal. A dual-energy X-ray absorptiometry (DEXA) scan of the left hip revealed osteopenia with a T-score of −2.0. Post-operative check X-rays revealed acceptable fixation (Figure 2). The mother and the baby were both discharged well.

**Figure 3:**
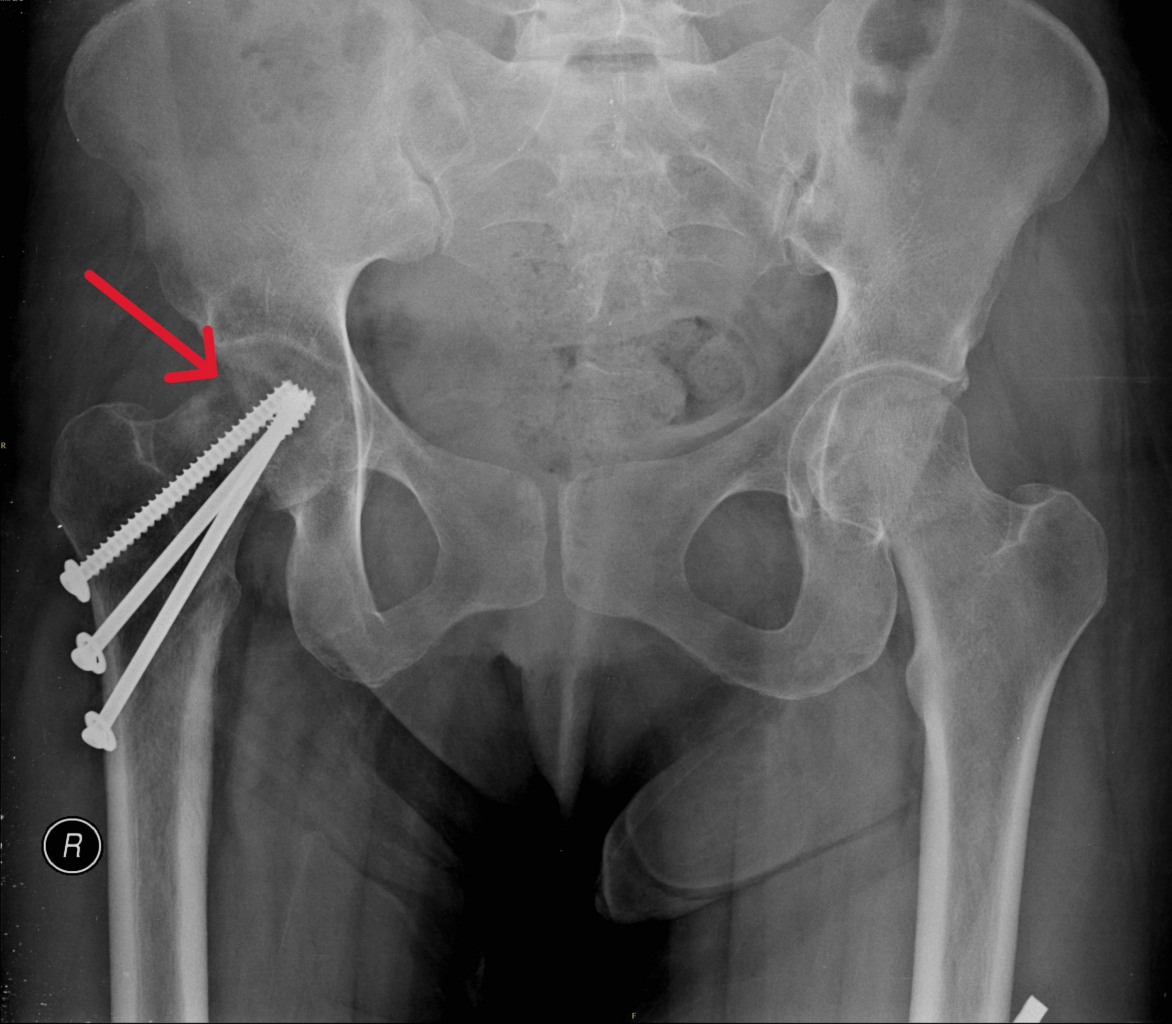
Pelvic X-ray at one-year post-op The red arrow shows the area of radiolucency at the previous fracture site.

**Figure 4:**
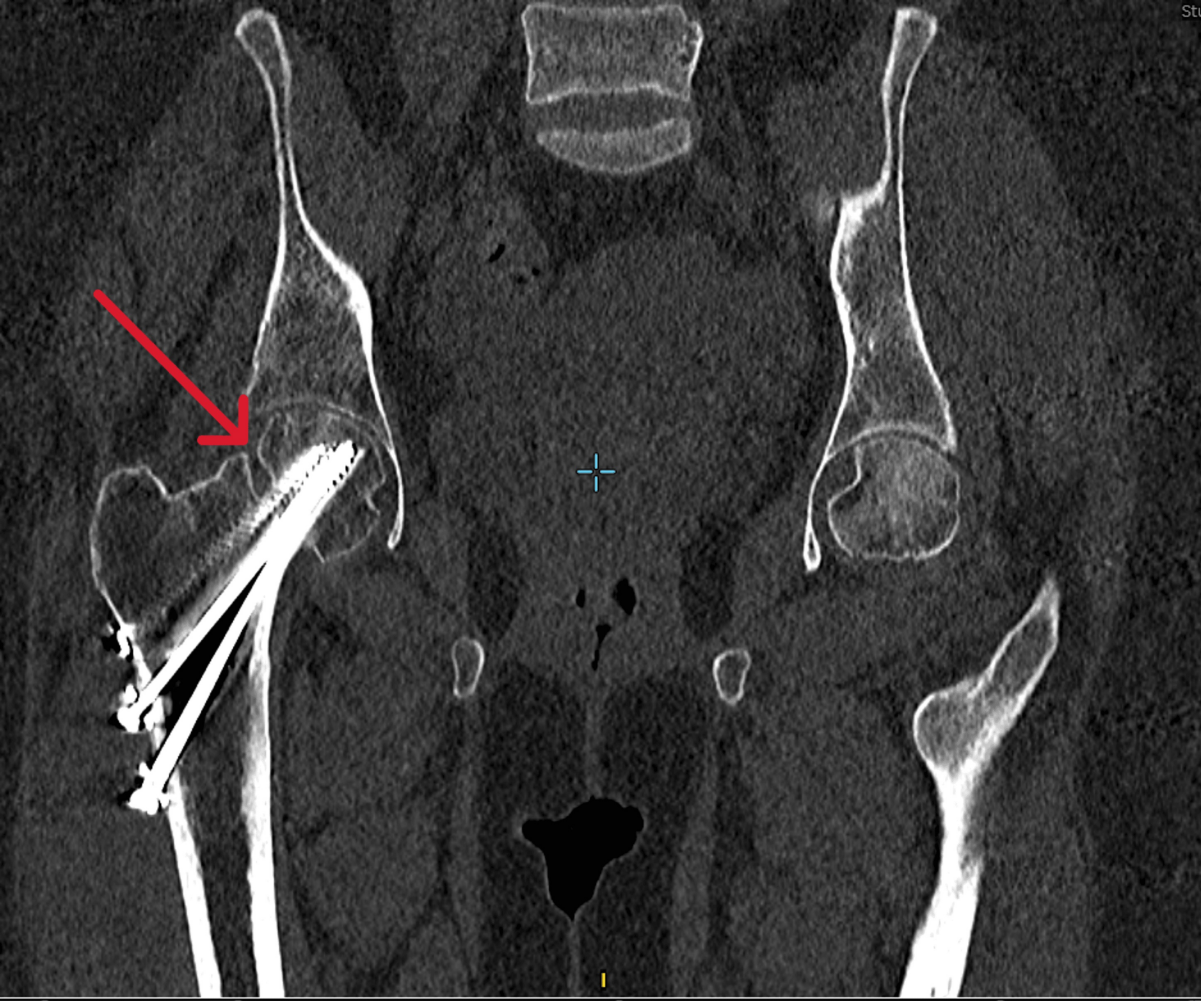
CT coronal view at one-year post-op The red arrow shows the lack of osseous bridging, along with cortical defects and well-defined fracture lines, confirming the diagnosis of non-union.

During follow-up at one year, X-rays showed radiolucency over the fracture site (Figure 3), and computed tomography (CT) of the right hip confirmed the diagnosis of non-union (Figure 4). Despite this complication, the patient remained asymptomatic for up to two years post-operatively and ambulated without assistive devices. Hip range of motion was normal except for some limitations in external and internal rotation. The patient was on regular surveillance of symptoms thereafter without any intervention being offered at the time.

## Discussion

TOH is a rare but important cause of hip pain and pathological fractures in pregnancy. It typically presents in the third trimester or early postpartum period and is more common in primigravida women. Clinically, TOH is a diagnosis of exclusion, meaning that other more severe conditions such as ONFH, fractures, malignancy, infection, and inflammatory arthritis must be ruled out [3].

The exact pathophysiology of TOH remains unclear. One of the widely accepted hypotheses is that venous congestion and increased intraosseous pressure in the femoral head lead to transient bone demineralisation, resulting in increased bone fragility [4]. In pregnancy, hormonal changes, calcium metabolism changes, mechanical pressure from the enlarging uterus, and decreased mobility also contribute to the development of TOH [3].

The case presented here highlights the diagnostic challenge of TOH, as the patient was initially misdiagnosed with pelvic girdle pain, which is more common in pregnancy. Pelvic girdle pain typically presents as lumbosacral pain and may radiate to the hips, often making it difficult to distinguish from TOH without appropriate imaging. The failure to restrict weight-bearing at the initial presentation likely contributed to the eventual fracture, a serious complication of TOH.

Magnetic resonance imaging (MRI) is the diagnostic modality of choice for TOH. MRI findings in TOH include diffuse bone marrow oedema, seen as hypointensity in T1-weighted sequences and hyperintensity in T2-weighted sequences [6,7]. MRI is relatively safe in pregnancy, especially in the second and third trimesters [8,9], and should be performed to confirm the diagnosis of TOH when clinical suspicion arises. Unfortunately, in this case, an MRI was not performed when the patient was presented with prodromal hip pain one month prior to the fracture. Furthermore, all patients with TOH show decreased bone mineral density (BMD) in the affected hip. This is consistent with the patient’s DEXA scan showing osteopenia, which reflects a reduction in bone density [10]. The decreased BMD is thought to be transient and reversible in TOH, with bone density returning to normal after delivery or within several months of conservative treatment.

Treatment for TOH is usually conservative, including restricted weight-bearing, physical therapy, and non-steroidal anti-inflammatory drugs (NSAIDs). There were case reports where complete resolution of TOH was noted after treatment with bisphosphonates [11]. However, bisphosphonates should be administered with caution in pregnant ladies in view of the harmful effects on both the mothers and foetuses [12]. On the other hand, when TOH leads to a femoral neck fracture, surgical intervention is required. The timing of surgery in cases of femoral neck fracture remains debatable. Some studies suggest that early surgical intervention reduces complications such as non-union and osteonecrosis, while others argue that fracture displacement, rather than the timing of surgery, plays a more significant role in the development of these complications [13,14]. In this case, the fracture was displaced, and the operation was done after the seventh day of admission, increasing the risk of osteonecrosis and non-union, as confirmed by a postoperative CT scan.

The timing of surgery in pregnant patients with femoral neck fractures also presents an additional challenge, as it requires careful consideration of both maternal and foetal well-being. In this case, the patient was at 33 weeks and four days of gestation, a crucial time for foetal lung maturity. The patient’s diagnosis of asymptomatic COVID-19 added complexity to her management. COVID-19 infection in pregnancy poses additional anaesthetic risks for both the mother and foetus. A multidisciplinary team, including obstetricians, paediatricians, orthopaedic surgeons, anaesthesiologists, and infectious disease specialists, was essential in deciding the optimal sequence and timing of both delivery and fracture fixation. The ideal timing of surgery was determined to be after the completion of quarantine for COVID-19 when the gestational age was more than 34 weeks, and the foetal lung was more developed from the corticosteroid treatment.

## Conclusions

TOH in pregnancy is a rare but self-limiting condition that can result in serious complications if not diagnosed early and treated properly. MRI should be considered for pregnant women with persistent hip pain, and weight-bearing restrictions should be enforced to prevent fractures. In cases of femoral neck fractures, multidisciplinary management is crucial, especially in complex scenarios involving prematurity and infections like COVID-19. Further research is needed to establish standardised guidelines for managing such cases.
